# Replaceable Jejunal Feeding Tubes in Severely Ill Children

**DOI:** 10.1155/2017/2090795

**Published:** 2017-01-23

**Authors:** Tabea Pang, Sergio B. Sesia, Stefan Holland-Cunz, Johannes Mayr

**Affiliations:** Department of Pediatric Surgery, University Children's Hospital Basel, Spitalstrasse 33, 4056 Basel, Switzerland

## Abstract

Long-term enteral nutrition in chronically ill, malnourished children represents a clinical challenge if adequate feeding via nasogastric or gastrostomy tubes fails. We evaluated the usefulness and complications of a new type of surgical jejunostomy that allows for easier positioning and replacement of the jejunal feeding tube in children. We surgically inserted replaceable jejunal feeding tubes (RJFT) connected to a guide thread which exited through a separate tiny opening of the abdominal wall. In a retrospective case series, we assessed the effectiveness and complications of this technique in severely ill children suffering from malnutrition and complex disorders. Three surgical complications occurred, and these were addressed by reoperation. Four children died from their severe chronic disorders within the study period. The RJFT permitted continuous enteral feeding and facilitated easy replacement of the tube. After the postoperative period, jejunal feeding by RJFT resulted in adequate weight gain. This feeding access represents an option for children in whom sufficient enteral nutrition by nasogastric tubes or gastrostomy proved impossible. Further studies are required to investigate the safety and effectiveness of this surgical technique in a larger case series.

## 1. Introduction

Long-term enteral nutrition results in better bowel regeneration, a lower rate of infective complications, and fewer side effects when compared to long-term parenteral nutrition [1, 2]. In addition, enteral nutrition prevents parenteral nutrition-associated liver disease (PNALD) [3]. Moreover, extravasation of parenteral nutrition solution can result in soft tissue damage [4], and insertion of intravenous lines is painful for children and incurs stress to caregivers and hospital staff [5].

Gastrostomy feeding has gained widespread acceptance for long-term enteral nutrition in children unable to eat or drink sufficient quantities. It allows for bolus feeding and ensures safer administration of enteral nutrients when compared to other types of long-term enteral nutrition techniques. However, in children suffering from neurologic impairment, severe gastroesophageal reflux disease (GERD), or disorders of gastric or esophageal motility, feeding by gastrostomy is frequently accompanied by complications such as recurrent aspiration, regurgitation, aerophagia, recurrent vomiting, and weight loss [6, 7]. Although fundoplication has gained widespread acceptance among pediatric surgeons in the treatment of severe GERD, the high rate of GERD recurrence and more frequent postoperative complications in neurologically impaired, malnourished children have made jejunostomy feeding an accepted alternative to establish long-term enteral feeding access in these children [7–10]. Several techniques for insertion of jejunal feeding tubes have been proposed, such as JET-PEG [11–13], fine-needle catheter jejunostomy, endoscopically guided, laparoscopically controlled, percutaneously inserted jejunal feeding tubes or buttons [8, 12–14], or insertion of jejunal feeding tubes by laparotomy or laparoscopy [14].

In the past years, several problems associated with long-term use of jejunal feeding tubes have been reported, such as dislocation or blockage of the feeding tube and small bowel obstruction. Changing a jejunal feeding tube and correct placement of a new feeding tube may be hampered by several complications, including incorrect placement, kinking, or recurrent dislocation of the tube [15–17].

The introduction of a new insertion technique for jejunal feeding tubes permitting easier tube replacement together with a safer two-point fixation technique of the feeding tube addressed the problems formerly encountered with the use of jejunal feeding tubes in neurologically impaired, malnourished children [18].

This single-center, retrospective case study aimed to describe the indications for jejunal feeding tube placement, postoperative complications, and weight gain observed in this group of critically ill children requiring mid-term or long-term enteral feeding access.

## 2. Patients and Methods

We conducted a retrospective, descriptive case study in patients who underwent jejunal feeding tube placement according to the surgical technique described by Schimpl et al. [18]. All children aged between 0 and 16 years who underwent jejunal tube placement between June 2005 and July 2011 were included in this investigation. We recorded patient demographics, main disorders of children, their medical and surgical histories, pre- and postoperative weight percentiles, type and number of postoperative complications, and nursing observations associated with jejunal tube feeding. The study protocol was approved by the ethical committee of Basel (protocol number: 2011/37).

## 3. Surgical Technique

**Figure 1:**
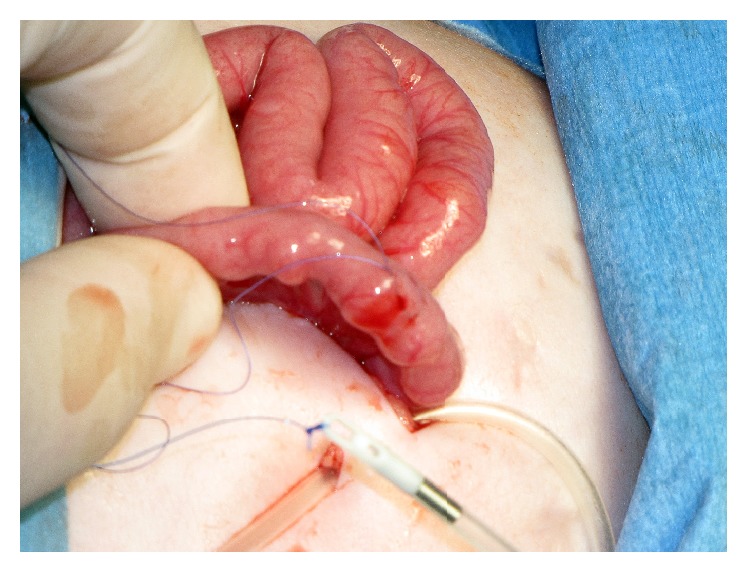
Placement of a purse string suture at antimesenteric aspect of the second jejunal loop. The feeding tube attached to a guide thread was introduced into the abdominal cavity (girl aged 2 months; patient 8).

**Figure 2:**
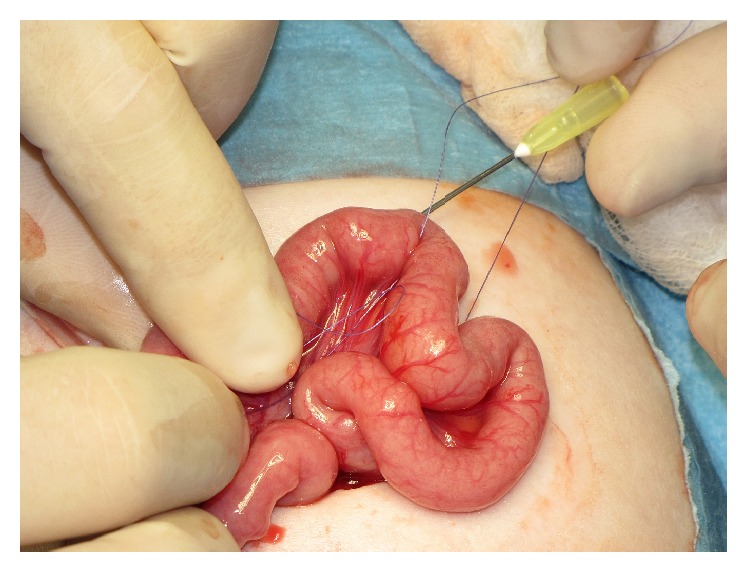
Insertion of the guide thread into the jejunal loop through the bore of a long injection needle (same patient as in Figure 1).

**Figure 3:**
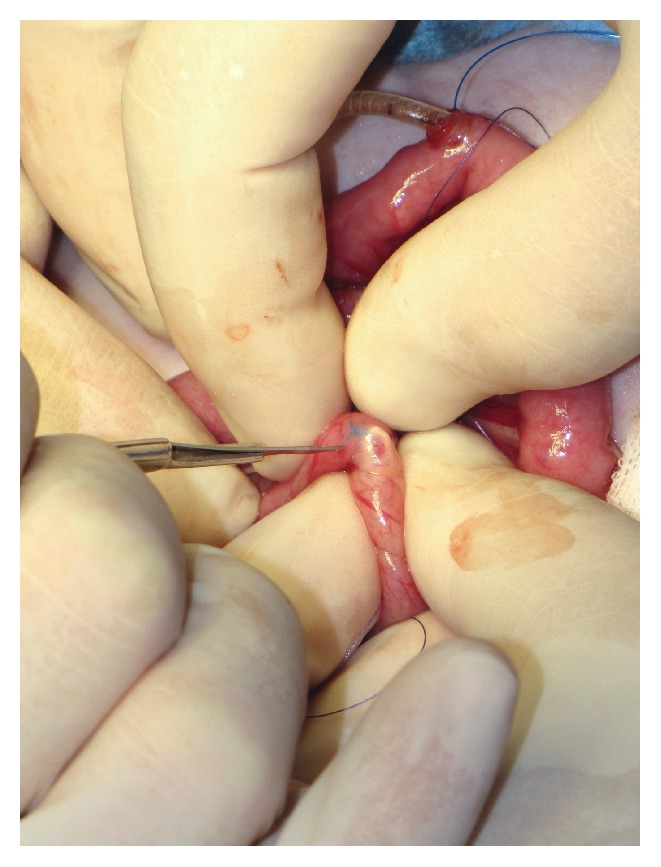
Because the small bowel in this very young patient (2 months) was too narrow to insert the needle over a distance of at least 8 cm without piercing the bowel wall inadvertently, we inserted the feeding tube equipped with a guide thread into the bowel lumen. An incision was made at the antimesenteric side of the jejunum loop at the planned exit site of the guide thread.

**Figure 4:**
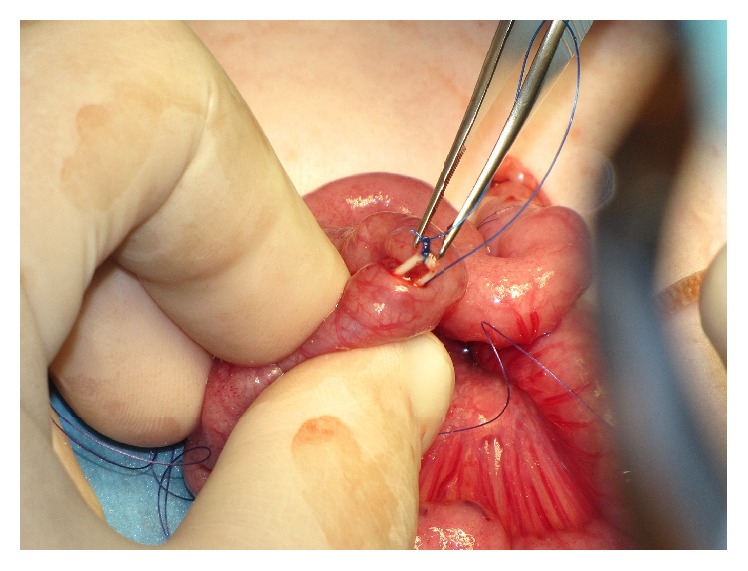
The guide thread was grasped and brought out through the abdominal wall using a needle.

**Figure 5:**
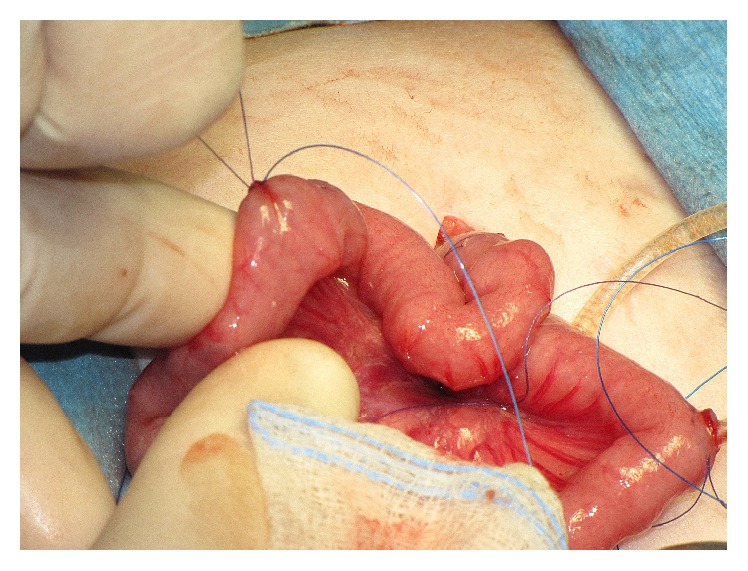
A suture was placed to close the opening of the bowel wall, and the suture was used to fix the bowel wall to the peritoneum of the abdominal wall.

**Figure 6:**
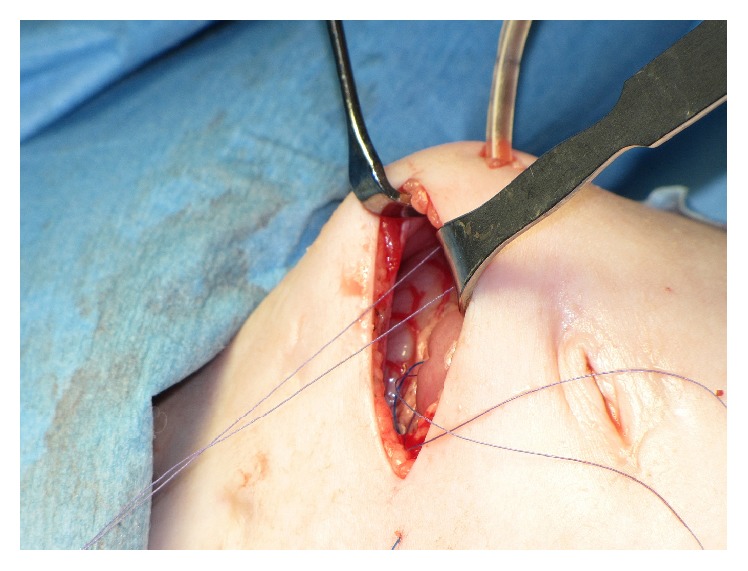
Both jejunal openings were sutured to the peritoneum of the abdominal wall. The guide thread exit site was placed to the right of the wound and the jejunostomy to the left.

After introduction of general anesthesia, children underwent surgical placement of an RJFT by a small transverse left upper abdominal incision (Figures 1, 2, 3, 4, 5, and 6). The chosen point of entry of the jejunal tube was at the second jejunal loop, approx. 15–20 cm from the ligament of Treitz. The exit point for the guide thread was 8–10 cm distally of the entry point. Both jejunal openings were secured with a purse string suture and fixed to the peritoneum of the abdominal wall using two absorbable stitches (Figure 6). The feeding tube (Freka® intestinal tube CH9 for PEG15; Fresenius Kabi, Schweiz AG, Stans, Switzerland) was attached to a 30 cm long 4-0 monofilament nonabsorbable thread for fixation and positioning of the tube tip within the jejunal loop. The guide thread was brought out through the abdominal wall and fixed to the abdominal wall at the epigastrium by applying adhesive tape. The laparotomy incision was closed, and jejunal tube feeding was initiated by slowly increasing the amount of nutrition solution infused continuously by a pump after a resting period of 6 h to 12 h [18].

## 4. Replacement of Jejunal Feeding Tube

**Figure 7:**
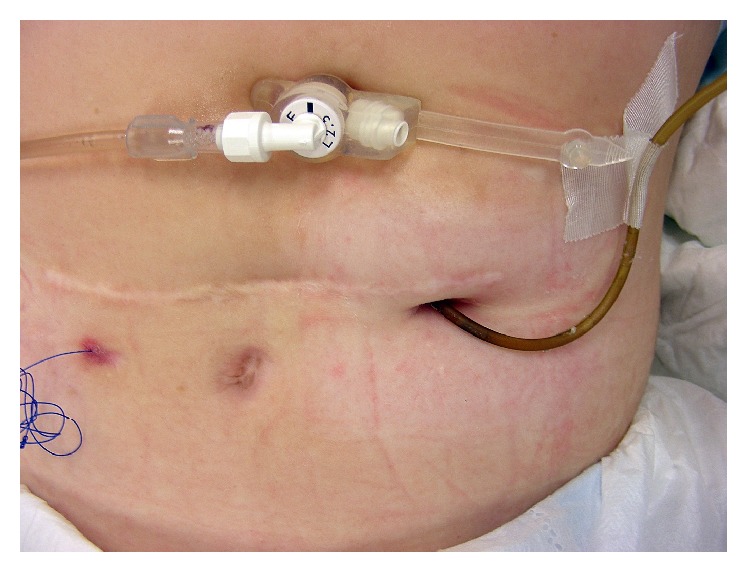
Photograph of a discolored jejunal feeding tube before tube exchange. Note the coiled guide thread to the left of the scar and gastrostomy button in the epigastrium.

We recommended replacement of the feeding tube at least every 6 months or when incrustations or discolorations of the feeding tube were observed (Figure 7). We applied anesthetic cream (Emla® Crème 5%, AstraZeneca, Zug, Switzerland) and an injectable anesthetic solution. A new monofilament nonabsorbable thread was sutured to the thread fixed to the tip of the feeding tube, and the feeding tube was withdrawn from the jejunostomy. A new feeding tube was sutured to the new thread, and the old thread and tube were discarded. The jejunal tube was reinserted into the jejunal loop, and the thread measuring 30 cm was coiled and fixed with adhesive tape to the abdominal skin surface close to the exit site of the thread. The new tube was fixed with one or two monofilament sutures and/or adhesive tape to the skin close to the point of entry of the tube. Parents and caregivers were instructed to replace the adhesive tapes at least once a week (or earlier if it became loose) and to apply gentle traction to the guide thread before fixing it to the skin surface. We advised parents and caregivers to apply continuous enteral feeds to prevent dumping syndrome.

## 5. Results

Eight children (6 girls, 2 boys) at a median age of 27 months (range: 2 months to 13 years) underwent laparotomy for surgical placement of an RJFT. All children suffered from severe malnutrition, defined by weight for age <3rd percentile and recurrent vomiting.

**Figure 8:**
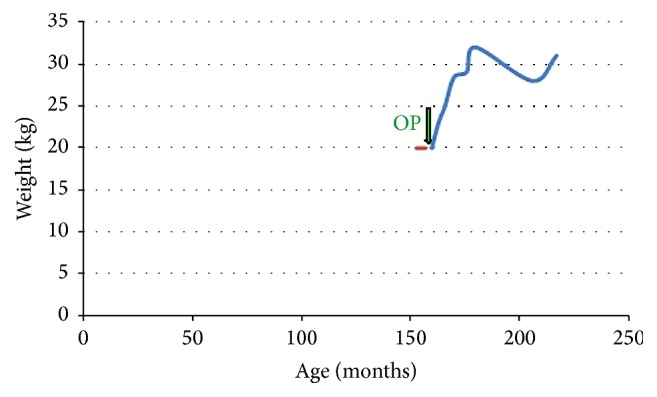
Weight development in patient 1 suffering from Wilkie syndrome [19].

In the first patient, a girl aged 13 years suffering from congenital selenoprotein-defective myopathy, Wilkie syndrome [19], severe scoliosis, hypoxic brain disorder, pulmonary hypertension, and respiratory insufficiency necessitating continuous ventilation therapy for 2 months, insertion of the RJFT allowed for jejunal feeding and weight gain (Figure 8). The main clinical symptom encountered in the postoperative period was a recurrent chronic abdominal pain syndrome and recurrent Clostridium difficile enterocolitis.

**Figure 9:**
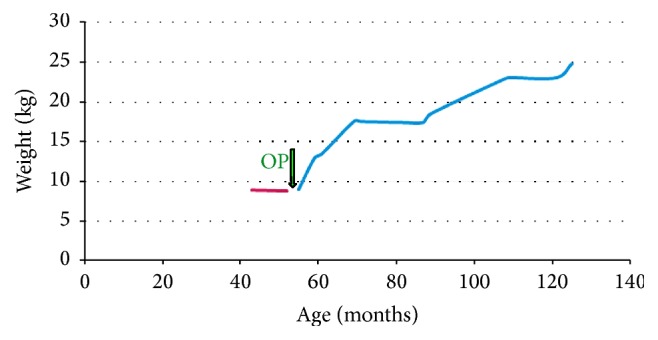
Weight development in patient 2 suffering from complex congenital malformation syndrome, respiratory insufficiency with ventilator dependency, recurrent aspirations, cerebral palsy, and epilepsy.

In patient 2, a boy aged 4.5 years suffering from a complex congenital malformation syndrome with bilateral cleft palate, neurogenic scoliosis and dislocation of the hip, respiratory insufficiency with ventilator dependency for a period of 2 months, recurrent aspirations, cerebral palsy, and epilepsy, insertion of the RJFT resulted in weight gain (Figure 9) and less frequent pneumonia episodes.

Patient 3, a 4.5-year-old boy, suffered from VACTERL syndrome and Fanconi anemia and underwent allogenic stem cell transplantation [20]. Graft-versus-host disease (GvHD) of the gastrointestinal tract with recurrent blood-stained vomiting occurred. Parenteral nutrition and immunosuppressive therapy were started, followed by insertion of an RJFT.

Patient 4, a 6-month-old girl, suffered from multiple congenital malformations, asplenia, psychomotoric retardation with generalized hypotonia, corpus callosum agenesis, and complex heart malformation complicated by an intracardial tumor. Impairment of laryngopharyngeal swallowing and inability to cough adequately resulted in recurrent aspiration episodes. Due to an absent sucking and swallowing reflex, neonatologists initiated tube feeding after birth. Within one year, the patient achieved a weight gain of 2.5 kg (from 10.0 kg to 12.5 kg).

In patient 5, a girl who underwent an operation for anaplastic ependymoma WHO grade III at the age of 5 months, occlusive hydrocephalus necessitated the placement of a ventriculoperitoneal shunt. We placed an RJFT to overcome the feeding problems associated with the neurological impairment characterized by dysphagia, swallowing dysfunction, hemiparesis, and facial nerve palsy. Because of a bacterial infection of the ventriculoperitoneal shunt device and influenza virus infection, we took down the jejunostomy 11 days after surgery and performed an external drainage of occlusive hydrocephalus. The patient died from tumor progression some days after removal of the RJFT.

Patient 6, a girl aged 3 years, suffered from progressive encephalopathy caused by intractable epilepsy, hypertrophic cardiomyopathy, pulmonary hypertension, central blindness, and neurogenic dislocation of the hip. Psychomotor retardation and recurrent aspirations complicated the clinical course, and treatment of epilepsy necessitated administration of a ketogenic diet. Because the girl was unable to swallow this diet, a nasogastric tube was placed, followed by percutaneous endoscopic gastrostomy placement (PEG). Feeding by PEG was poorly tolerated. Recurrent vomiting, massive tracheobronchial mucus secretion, recurrent aspiration episodes, and malnutrition complicated the clinical course. An RJFT was placed, which made adequate enteral nutrition and administration of the ketogenic diet possible. We enrolled the child in an outpatient enteral home-feeding program. The child died from progressive encephalopathy 6 months after the RJFT insertion.

**Figure 10:**
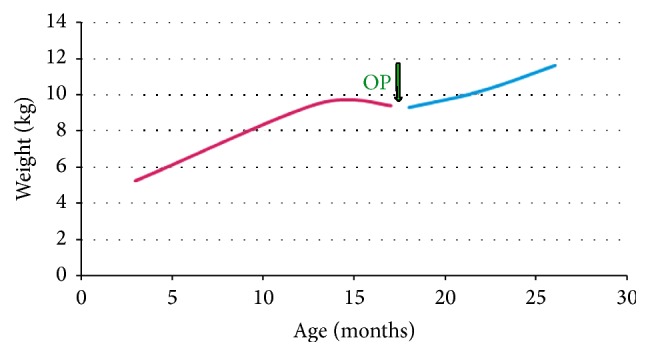
Weight development in patient 7 suffering from progressive encephalopathy caused by intractable epilepsy.

Patient 7, the younger (15 months) sister of patient 6, suffered from the same disorders as her sister. Her main clinical problems comprised intractable epilepsy, recurrent vomiting episodes complicated by hematemesis, and insufficient weight gain (Figure 10).

**Figure 11:**
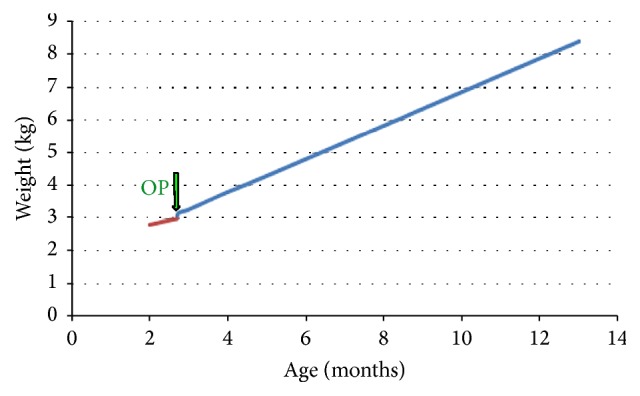
Weight development in patient 8 suffering from a microdeletion 22q11 syndromic disorder with cardiac malformation. After 3 months of RJFT use, the tube was removed, and the girl continued to grow well on oral feeding, with a weight for age at the 30th percentile.

Patient 8, a 2-month old girl, suffered from a microdeletion 22q11-syndromic disorder with cardiac malformation. Enteral nutrition by nasogastric tube was started due to pronounced swallowing dysfunction, absent sucking reflex, and aspiration episodes. After 4 weeks, the swallowing ability of the girl improved. Because an upper gastroesophageal contrast study revealed adequate swallowing with absent gastroesophageal reflux episodes, oral feeding was started and was well tolerated. After 3 months, the RJFT was removed and the girl continued to grow well with a weight for age at the 30th percentile (Figure 11).

## 6. Complications of RJFT Placement

Table 1 shows the complications after RJFT placement. In the first patient, the thread connected to the tip of the feeding tube used for correct placement of the tube within the jejunal loop became loose as the adhesive tape which held it in place detached itself from the skin. A long part of the thread ran through the abdominal cavity and caused small bowel obstruction. At the revision surgery, we sutured the exit site of the thread at the antimesenteric boarder of the jejunum loop to the peritoneum of the abdominal wall. We applied this type of sutures in all further patients undergoing RJFT placement.

In patient 2, partial volvulus occurred 7 months after insertion of the RJFT when small bowel loops and colon became entrapped in a large congenital Morgagni hernia. Torsion occurred around the jejunostomy. We took down the jejunostomy by open surgery, reduced the volvulus, repaired the hernia, and placed a new RJFT.

In patient 3, who suffered from Fanconi anemia and GvHD after allogenic stem cell transplantation, enteral feeding was poorly tolerated due to bowel dysfunction related to Fanconi anemia and gastrointestinal GvHD. After several unsuccessful attempts to increase the volume of enteral feeding solution, we removed the feeding tube. However, the jejunostomy did not close spontaneously, and bacterial infection of the abdominal wall surrounding the jejunostomy occurred. The immunocompromised child died 3 weeks after the operation.

In patient 4, bacterial infection of the ventriculoperitoneal shunt occurred after insertion of the RJFT. The RJFT was removed after 11 days, and the ventriculoperitoneal shunt was exteriorized. Enteral feeding using a nasogastric tube was started again. Microbiological examination of the liquor revealed growth of* Enterobacter cloacae*. Due to incomplete resection of the brain tumor, the treatment situation was considered palliative, and the patient died from tumor progression.

In patient 6, the RJFT was used for 5 months. The feeding tube was replaced once because of massive incrustations from medications administered through the tube. The girl died from intractable epileptic seizures, hypertrophic cardiomyopathy, pulmonary hypertension, and pneumonia. An underlying autosomal recessive mitochondrial disorder was thought to be the cause of the condition of this girl and her sister (patient 7).

**Figure 12:**
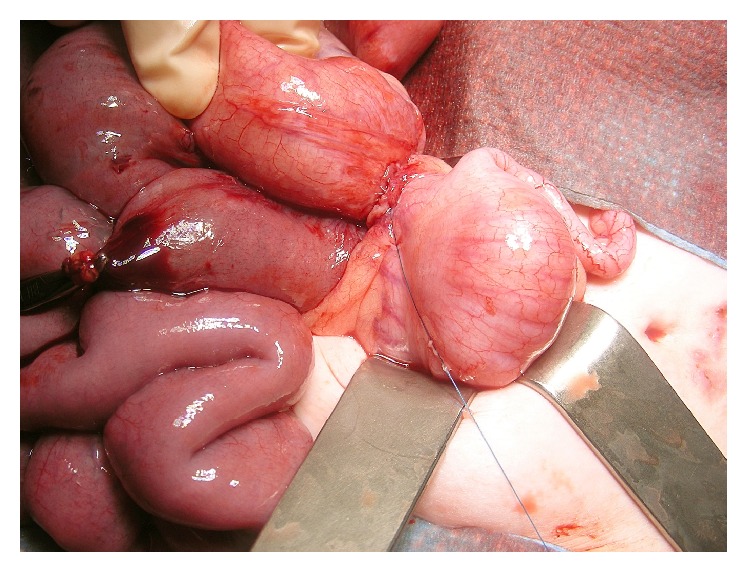
Acute small bowel obstruction in a girl aged 20 months. Bowel obstruction was caused by a loose guide thread and fibrous band crossing the terminal ileum. This complication occurred 18 months after RJFT insertion and was managed by resection of the fibrous band and guide thread (patient 7).

In patient 7, the younger sister of patient 6, the RJFT was used for 5.2 years. Eighteen months after surgical feeding tube placement, acute small bowel obstruction occurred. Bowel obstruction was caused by entrapment of the ileocecal bowel segment in a loop of the guide thread. Small bowel obstruction was repaired surgically (Figure 12).

**Figure 13:**
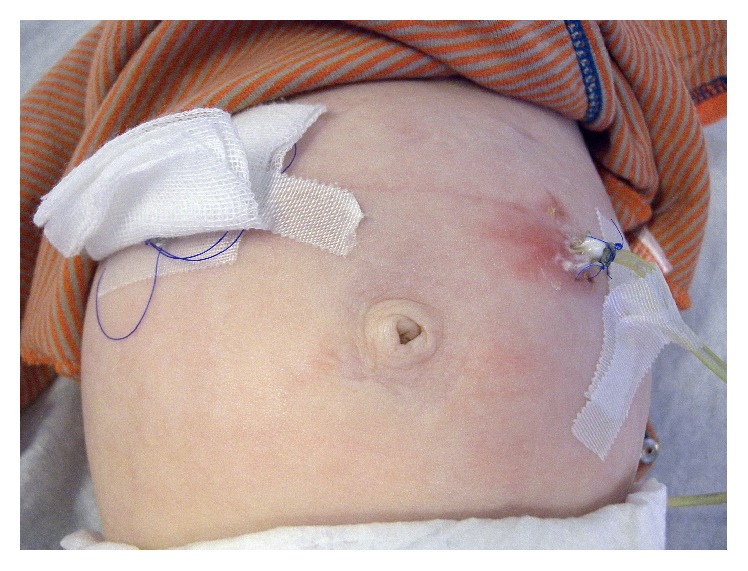
Local skin infection at the site of the jejunostomy. This infection was treated with antibiotic ointment and enterally administered antibiotics.

In this child, 3 episodes of skin infection occurred at the entrance site of the jejunal feeding tube within the regular follow-up period (Figure 13). These superficial skin infections were managed with antiseptic ointments and enteral antibiotics. Ten months after insertion of the feeding tube, the child developed pneumonia after aspiration of gastric contents. The RJFT was replaced uneventfully at intervals of 6 months.

In patient 8, RJFT use was monitored for a period of 3 months only. The tube underwent dislocation once, which was corrected by repositioning and fixation of the tube and thread using adhesive tape. An episode of local skin infection was managed with antibiotic ointments and systemic antibiotics.

## 7. Follow-Up of Patients

Long-term follow-up results beyond the study period are currently available for 4 patients. Patient 1 has been managed by enteral RJFT feeding for 11.8 years and patient 7 for 5.2 years. However, in patient 7 the RJFT was changed into a jejunal tube without guide thread during the surgical intervention for small bowel obstruction 18 months after insertion of the RJFT. Patient 2 died from his underlying disorders after 9.5 years of enteral RJFT feeding. Patient 8 has been eating normally since RJFT removal 6 years ago. None of these patients underwent any further surgical interventions. We observed further weight gain in all patients within the long-term follow-up period. Patients 3, 4, 5, and 6 died from their underlying disorders within the study period.

## 8. Discussion

Because of the retrospective nature of this single-center case series and the variable disorders of the children, the results must be interpreted with caution. All children suffered from disorders complicated by severe malnutrition and recurrent vomiting. In two children, a ketogenic diet was not tolerated when administered by the oral route or nasogastric tube, and insertion of an RJFT allowed for successful administration of the ketogenic diet.

When long-term feeding via gastrostomy is poorly tolerated because of recurrent aspiration, regurgitation, or gastroesophageal reflux disease jejunal application of enteral feedings is a promising option to facilitate enteral nutrition in children. Percutaneous gastrojejunostomy with transgastric jejunal insertion of a feeding tube using a neonatoscope and guidewire permits for a fast and safe insertion of a jejunal feeding tube with minimal exposure of the child to ionizing radiation. However, Michaud et al. reported that, in 27 patients followed up for a median period of 5.5 months, 31 tube dislodgements, 16 tube obstructions, 7 leakages around the tube, 6 internal ballon ruptures, and 1 intussusception occurred [17]. Additionally, 11 of 27 children required surgery, and the authors conclude that the high rate of complications and tube replacements limits the use of this jejunal feeding strategy [17]. Raval and Phillips reported that in a group of 20 children who did not tolerate gastrostomy feeding 14 children underwent image-guided jejunal feeding tube placement [20]. Half of these children ultimately underwent Roux-en-Y jejunostomy placements [20]. Image-guided jejunal feeding tube placement patients required 4.6 fluoroscopy-guided feeding tube revisions per year [20], which exposed them to a considerable amount of ionizing radiation.

Transgastric feeding tubes dislodge easily, and Kaplan et al. noted a rate of 84% jejunal feeding tube malfunctions, caused by tube dislodgements, tube obstructions, and leakages around the tube at a mean interval of 39 days after placement of the tube [21].

However, there are some reasons, why image-guided feeding tube placement represents a well-accepted first choice for jejunal feeding tube placement in children. These include a less invasive procedure, which can be performed in sedation in contrast to general anesthesia and easy discontinuation of the tube after treatment once the child no longer requires jejunal feeding [20].

In a 2-center study from Leeds and Manchester on the limitations and clinical usefulness of gastrojejunal feeding tubes in 18 children (12 of these suffered from neurological impairment) followed up for a median time interval of 10 months, the authors reported 65 tube related complications in 14 children [22]. Jejunal tube dislodgement was the most frequently observed complication, and 4 children suffered from recurrent aspiration, bilious vomiting, and diarrhea after onset of jejunal tube feeding [22]. In our study, 6 of 8 children suffered from neurological impairment. To the best of our knowledge, there exist no relevant systematic reviews or prospectively randomized controlled trials on gastrostomy or jejunostomy feeding in children with cerebral palsy [23].

Comparing our complication rate of surgical RJFT insertion to the complication rate of the “Omega”-jejunostomy tube technique, we noted a lower rate of complications for the second [24]. However, the small group of children we treated was younger (median age: 27 months) when compared to the patients treated by Schlager et al. with a “Omega” jejunostomy and button placement (median age at surgery: 11 years) [24]. Due to the low number of patients in both studies, further studies are required to confirm these results. We hypothesize that thread related complications observed in older children might be reduced by insertion of a small low profile button device into the jejunostomy after maturation of the jejunostomy, to avoid long-term use of the thread in older children. Further studies are required to evaluate this hypothesis.

Compared to esophagogastric disconnect, which is used when fundoplication failed and gastric feeding is no longer an option, the insertion of a RJFT is less technically demanding [24, 25].

Smith and Soucy followed up 57 pediatric patients in whom 64 surgical jejunostomies were placed [15]. They found a mean duration of jejunostomy use of 1.1 ± 2.4 years and an overall complication rate of 37.5% including 21.9% major complications, which is similar to our findings in neurologically impaired children. We agree with Smith and Soucy that the benefits of long-term usage of surgical jejunostomies outweigh the risks for most patients perhaps those who are neurologically impaired or suffer from intractable seizures [15].

The children we were able to follow up for a prolonged period of time underwent weight gain after surgical insertion of the RJFT. Caregivers also reported that the episodes of vomiting occurred less frequently and were less severe. Parents and caregivers were satisfied with the handling of the RJFT, although continuous administration of enteral nutrition solution was required. We discouraged parents and caregivers to apply bolus feeding due to the risk of dumping syndrome.

The RJFT was easily exchanged in the outpatient office, and application of lidocaine/prilocaine cream 5% (Emla cream® 5%, AstraZeneca, Zug, Switzerland) was considered helpful to avoid pain at the jejunostomy and exit site of the guide thread. However, in an older girl who was highly sensitive to pain, we exchanged the feeding tube under anesthesia or conscious sedation. We recommend to exchange the feeding tube at 6-month intervals and to rinse the tube with a small volume of water after every administration of enteral nutrition solution to avoid incrustation of the narrow tube. We discourage the routine administration of drugs using the jejunal tube to avoid blockage of the 9 CH tube lumen.

The immunocompromised child suffering from Fanconi anemia developed a jejunal fistula after removal of the feeding tube (Table 1). We noted that jejunal feeding was not adequately tolerated in this child and hypothesized that the impaired absorptive, digestive, and regenerative capacity of the bowel mucosa may be responsible for this phenomenon in children suffering from Fanconi anemia [27]. This situation may worsen when allogenic stem cell transplantation (STX) results in GvHD of the bowel mucosa [27]. We therefore do no longer recommend applying this feeding technique in children suffering from Fanconi anemia after allogenic STX.

## 9. Conclusions

The use of RJFT facilitated long-term jejunal feeding access in chronically ill children suffering from severe malnutrition, complex chronic disorders, and recurrent vomiting in whom feeding by nasogastric tube or PEG had failed. The most relevant surgical complication observed with RJFT placement was acute small bowel obstruction caused by the guide thread. Due to the limited number of children, the results of this retrospective study on the use of RJFT should be interpreted with caution. Further studies are required to investigate the effectiveness and safety of this feeding technique in children.

## Figures and Tables

**Table 1:** Complications after RJFT placement.

Postoperative complications	*n*
Complications necessitating surgical intervention (volvulus, ventriculoperitoneal shunt infection, or small bowel obstruction)	3
Guide thread erroneously cut off^*∗*^	1
Local skin infection at jejunostomy	5
Blockage of feeding tube due to incrustations	2
Dysfunction caused by kinking of feeding tube	2
Persistent jejunal fistula in an immunocompromised child suffering from Fanconi anemia and GvHD	1

*Total *	*14 *

^*∗*^In one child (patient 7), a nurse erroneously cut off the guide thread and the jejunal tube fell off. We inserted a new tube connected to a new guide thread by an endoscopically assisted procedure [26].
